# Neural state changes during movie watching relate to episodic memory in younger and older adults

**DOI:** 10.1093/cercor/bhaf114

**Published:** 2025-05-19

**Authors:** Sarah E Henderson, Djamari Oetringer, Linda Geerligs, Karen L Campbell

**Affiliations:** Department of Psychology, Brock University, 1812 Sir Isaac Brock Way, L2S 3A1 St. Catharines, ON, Canada; Donders Institute for Brain, Cognition and Behaviour, Radboud University Nijmegen, Thomas van Aquinostraat 4, 6525 GD, Nijmegen, Netherlands; Donders Institute for Brain, Cognition and Behaviour, Radboud University Nijmegen, Thomas van Aquinostraat 4, 6525 GD, Nijmegen, Netherlands; Department of Psychology, Brock University, 1812 Sir Isaac Brock Way, L2S 3A1 St. Catharines, ON, Canada

**Keywords:** neural states, event segmentation, episodic memory, aging, EEG

## Abstract

Event segmentation is a key feature underlying the ability to remember real-life occurrences. At the neural level, event boundaries have been shown to align with boundaries between neural states—stable patterns of brain activity maintained over time. These neural states provide a valuable window into the neural underpinnings of event perception. To investigate how neural state boundaries relate to memory across the lifespan, we used the data-driven Greedy State Boundary Search method to implicitly identify neural state changes in younger and older adults’ electroencephalography data during movie watching. Memory for the movie was tested and related to (1) neural state correspondence across individuals and (2) the degree to which the pattern of activity changes at boundaries. Neural state boundaries significantly aligned across people, but did not differ with age nor relate to memory. The degree of change at neural state boundaries also did not differ with age, but was positively related to memory for the movie. These findings suggest that age differences in the perception of naturalistic events may be less pronounced than previously thought, at least when measured implicitly, and that greater distinction between successive neural states relates to better memory for one’s experiences regardless of age.

## Introduction

### Event perception

While we experience everyday life as a continuous stream of information, we tend to perceive this stream as a series of events, with shorter event units nested within larger ones (eg the larger event of making dinner could consist of chopping vegetables, sautéing them, and adding stock). Recent work on event cognition suggests that the segmentation of continuous experience into meaningful event units influences our understanding (Zacks et al. 2001) and recall of events (Flores et al. 2017; Heusser et al. 2018), with greater alignment in event boundaries across participants related to better memory (for a recent review, see Zacks, 2020). Event boundaries tend to be perceived whenever we experience shifts in location (Radvansky and Copeland 2006; Horner et al. 2016; Brunec et al. 2020), time (Zwaan 1996; Speer and Zacks 2005; Ezzyat and Davachi 2011), or goals (Polyn et al. 2009; Wang and Egner 2022). Event segmentation itself seems to occur without intention, while the processing that occurs at event boundaries, including the refreshing of working memory, places demands on attentional control (Zacks et al. 2007). Indeed, it appears that the identification of event boundaries triggers a cascade of activity that influences memory encoding as reflected by memory-related medial temporal lobe activation surrounding boundaries (Ben-Yakov and Henson 2018; Reagh et al. 2020; Zheng et al. 2022; Barnett et al. 2024).

### Evidence for age differences in event segmentation

Given its apparent role in long-term memory formation, deficits in event segmentation have been posited as a potential cause of age differences in episodic memory. In support of this notion, some work has shown that older adults differ in their perception of event boundaries. During explicit boundary detection tasks, in which participants are asked to report (usually by pressing a button) when they believe that one “meaningful unit” has ended and another has begun, older adults have been shown to be more idiosyncratic in where they place boundaries (Kurby and Zacks 2011). As more normative segmentation on these tasks typically predicts better memory performance (Zacks et al. 2006; Bailey et al. 2013), older adults’ more idiosyncratic event segmentation may contribute to their often poorer episodic memory. These results are consistent with functional magnetic resonance imaging (fMRI) findings suggesting that older adults are more idiosyncratic in their processing of naturalistic stimuli (ie intersubject synchrony declines with age during movie watching; Campbell et al. 2015; Geerligs et al. 2018). This suggests a role for age differences in the perception of event boundaries in age-related memory deficits. However, under implicit conditions, event boundary perception seems to be less affected by age (Magliano et al. 2012; Kurby et al. 2014). For instance, older and younger adults show similarly slowed reading times at narrative transitions (Morrow et al. 1997; Radvansky and Curiel 1998; Stine-Morrow et al. 2001), similar increases in pupil diameter and gaze synchrony around event boundaries (Davis et al. 2021; Smith et al. 2023), and similarly reduced working memory performance following a change in events (eg reduced ability to recall an item from working memory after passing through a doorway; Radvansky and Curiel 1998). Furthermore, neural activation in segmentation-related areas is preserved in older adults, despite age differences in explicit boundary identification (Kurby and Zacks 2018). Age differences in event segmentation therefore seem to be most apparent on explicit tests and less pronounced when event segmentation is measured indirectly. Thus, the relationship between event perception, as measured by effortful segmentation tasks, and event memory may be partially attributable to their shared reliance on attentional control and other executive functions that are subject to considerable individual differences and typically decline with age.

The lack of age difference on implicit tests makes theoretical sense given that boundary detection relies largely on knowledge, with differences in boundary detection related to the level of familiarity or expertise one has with a task or event (Bläsing 2015; Smith et al. 2020; Newberry et al. 2021; Pförtner and Hristova 2024). While fluid abilities such as attentional control are known to decline with age, crystallized intelligence (ie knowledge) is preserved or even increased with advancing age (Horn and Cattell 1967; Umanath and Marsh 2014). The dependence of event segmentation on knowledge fits with common theories of event boundary detection as relying on prediction error (ie an event boundary is perceived when one's predictions fail; Zacks et al. 2007), and on learned event types or schemas (eg we know the basic steps involved in doing laundry; Shin and DuBrow 2021). Together, these findings suggest that the detection of event boundaries does not differ with age when tested implicitly (with the caveat that boundary agreement may differ when age groups differ in knowledge about the stimuli; Smith et al. 2020), but the cognitive processes triggered by event boundaries (ie working memory updating, memory consolidation) may differ with age.

### Aging and inhibition

A common observation in the aging literature is that older adults have relative impairments in attentional control, particularly in the inhibitory function of attention (Hasher et al. 2007; Campbell et al. 2020). This results in a reduced ability to delete no-longer-relevant information, such that older adults maintain items in working memory after they have become irrelevant (Scullin et al. 2011; Higgins et al. 2020). This failure to inhibit previously relevant information has also been shown to influence long-term memory, with older adults forming more erroneous associations between items from temporally adjacent pairs in a paired associates task (Campbell et al. 2014). Weeks et al. (2020) provided neural evidence of this age-related inhibitory deficit, showing that in a working memory task involving displays of four items from two categories (ie faces and places), older adults showed sustained neural representation of the to-be-forgotten category during the delay period after being told which category would be tested. Furthermore, better decoding of the irrelevant category during the delay predicted worse memory for the relevant items, suggesting that failure to delete irrelevant items from working memory interferes with memory for relevant information.

In recent behavioral work, we showed that this maintenance of irrelevant information in working memory may contribute to age differences in long-term event memory (Henderson and Campbell 2023). We used a cued recall task designed to test the degree of binding within versus between neighboring events which was adapted from Ezzyat and Davachi (2011). After having watched a movie, participants were given cues that came from either the middle of events (within-event cues) or just before an event boundary (between-event cues) and were asked to report what happened next in the movie. In general, participants showed better recall for within-event cues, reflecting the relatively stronger associations formed within events compared to those formed between events (Ezzyat and Davachi 2011). However, low-performing older adults (those who fell below the median on overall performance) showed a smaller benefit for within-event cues, suggesting that low-performing older adults form relatively stronger associations across boundaries (Henderson and Campbell 2023). These same older adults also did worse on a separate working memory updating task, suggesting that low-performing older adults may carry information with them across event boundaries and this affects overall memory for the events. In contrast, high-performing older adults showed better recall for within- than between-event cues, similar to that observed in younger adults, suggesting that better long-term memory relies on the formation of distinct events at encoding.

### Neural states as a marker of event perception

Though relatively less is known about the neural underpinnings of event segmentation, there is some consensus regarding the regions typically involved. Generally, evoked responses to event boundaries are observed in default network regions, including the precuneus and medial visual cortex (Speer et al. 2007; Zacks et al. 2010; Kurby and Zacks 2018), and the medial temporal lobes (Ben-Yakov and Henson 2018; Reagh et al. 2020). These likely reflect the updating of event models (Ezzyat and Davachi 2011) and the encoding of events in long-term memory, respectively (Ben-Yakov and Henson 2018). However, much like behavioral studies of event boundaries, previous studies of evoked neural responses to event boundaries typically relied on event boundaries identified by a separate, explicit event segmentation task (eg Ben-Yakov and Henson 2018). In recent years, there has been increased interest in techniques to segment continuous neural data and identify underlying neural states in a data-driven matter. In the first study to develop this approach (Baldassano et al. 2017), the authors applied a Hidden Markov Model (HMM)–based technique to fMRI data collected while participants watched a movie. This method identifies the times in the data when a region of interest transitions from one relatively stable pattern of activity (ie neural state) to another. This method can be repeated across the brain using a traveling searchlight method to identify the state boundaries and durations that are unique to different regions. These states tend to follow a temporal hierarchy (Baldassano et al. 2017; Geerligs et al. 2022) that is consistent with the brain’s information processing hierarchy (Hasson et al. 2008; Lerner et al. 2011), with shorter states in perceptual regions and longer states in higher-order regions. At the group level, these states have been shown to overlap across brain regions and importantly, to correspond with behaviorally derived boundaries across the cortical hierarchy, particularly with longer states on the time scale of tens of seconds over the frontal portion of the cortex (Baldassano et al. 2017; Geerligs et al. 2022).

While these neural state methods were developed for use with fMRI, they have also been successfully applied to electroencephalography (EEG) data (Silva et al. 2019; Mariola et al. 2022), which has better temporal resolution but worse spatial specificity. Though previous work has suggested that group-level data are required for stable output from both HMM-based neural state segmentation (Baldassano et al. 2017) and an alternative Greedy State Boundary Search (GSBS; Geerligs et al. 2021) method, emerging evidence suggests that stable states may be derived from individual subject data (Betzel et al. 2022; Sava-Segal et al. 2023; Wilford et al. 2024). By studying neural states at the individual level, it becomes possible to investigate normative segmentation (ie correspondence of neural states across people) with an implicit measure of segmentation and to study its relation to individual differences in age and memory.

### Current study

The main aim of the current study was to use neural state segmentation to determine the unique contributions of normative segmentation and relative boundary strength to age differences in memory for the movie. To accomplish this, older and younger adults viewed a shortened version of Alfred Hitchcock’s *Bang You’re Dead* while their neural activity was recorded with EEG. Participants then completed cued and free recall tests for the movie in the same manner as Davis et al. (2021). While most work has used group-averaged neural data to identify neural states during movie watching, here we take the novel approach of applying the GSBS segmentation algorithm (Geerligs et al. 2021) to each individual’s EEG data to determine the location of boundaries within each individual. We first assessed the validity of this metric by comparing individual boundary locations to the group average boundaries. As explicit event boundary segmentation has been shown to overlap across individuals, we expect that the correlation between group and individual neural state boundaries should be greater than what would be expected by chance. Similarly, previous work applying GSBS in fMRI (Geerligs et al. 2021, 2022) has shown significant overlap between neural state boundaries and behaviorally identified event boundaries. Thus, in addition to assessing the similarity in boundary location between individuals, we also tested whether there is significant overlap between the boundaries identified using individual-level GSBS and boundaries identified by a separate group of participants on an explicit event segmentation task. We then investigated the ability to predict memory performance from one’s correspondence to the group, an approach that is conceptually similar to previous behavioral work investigating the role of event segmentation agreement in memory performance. Our hypothesis was that older and younger adults should not differ on this implicit measure of event segmentation, but one’s correspondence to the group may relate to subsequent memory for the movie (eg Hasson et al. 2004; Zacks et al. 2006; Davis et al. 2021). We next tested whether the relative strength of neural state changes, as measured by the change in the pattern of neural activity around each individual’s boundaries, predicts memory performance. Here, we hypothesized that younger adults would show a greater change in the pattern of activity at boundaries than older adults and for more distinct boundaries to relate to better memory for the movie (Gold et al. 2017; Henderson and Campbell 2023).

## Methods

### Participants

Participants included 33 older (61 to 82 years) and 33 younger adults (18 to 27 years). This sample size was based on other similar aging and EEG studies (eg Strunk and Duarte 2019; Hellerstedt et al. 2021). Of these, five younger adults and two older adults were excluded for incomplete EEG or memory data. An additional three older adults were excluded for scoring <23 on the Montreal Cognitive Assessment, a well-validated neuropsychological test used for the detection of mild cognitive impairment (MoCA; Carson et al. 2018; Nasreddine et al. 2005), and two were excluded for reporting issues understanding the movie. This resulted in a final sample of 28 younger (*M*_age_ = 20.2, *SD* = 2.89; 18 female) and 26 older (*M*_age_ = 69.1, *SD* = 6.03; 16 female) adults. Older adult participants were invited to participate from a community database consisting of individuals recruited mainly through community events and local advertising and were paid $10/h for their participation. Younger adults were university students and received course credit for their participation. Older adults had more years of education (*P* < 0.001; older adults: *M* = 18.1, *SD* = 5.05; younger adults: *M* = 14.0, *SD* = 1.93) and higher Shipley vocabulary scores on average (*P* < 0.001; older adults: *M* = 35.1, *SD* = 4.80; younger adults: *M* = 29.0, *SD* = 3.62; maximum possible score is 40; Shipley 1940).

### Stimuli

The main stimulus was an 8-min version of Alfred Hitchcock’s *Bang You’re Dead*. This video has been widely used in previous research including in the Cambridge Centre for Aging and Neuroscience study (Cam-CAN; Shafto et al. 2014). For the cued recall task, scene changes (ie points in the movie when the scene faded to black or transitioned to a new time or location) were used as an approximation of event boundaries for cue creation. We extracted short clips from either the middle of the scene (within-event cues) or just before a scene cut (between-event cues). A total of 20 clips were used (10 within, 10 between; *M*_between_ = 5.60s, *SD*_between_ = 1.43 s; *M*_within_ = 5.30s, *SD*_within_ = 0.95 s). The duration of clips did not significantly differ between conditions, *t*(18) = 0.55, *P* = 0.587. We have used these clips previously to test cued recall within and between events for this movie (Davis et al. 2021).

### Electroencephalography

A 128-channel Active Two BioSemi system with common-mode-sense (CMS)/driven-right-leg (DRL) referencing was used to record scalp-level EEG with a sampling rate of 512 Hz. The recordings were made in a dimly lit, electrically shielded room. The videos were presented on a screen 60 cm from the participant. The audio was presented through ER2 headphones with 30-dB external noise exclusion and 70-dB isolation between ears. The volume was individually adjusted to a comfortable level using a short video clip prior to the main movie.

### Procedure

The study was approved by the Research Ethics Board at Brock University (File number: 17-024). All tasks were completed with the understanding and written consent of each participant. The behavioral procedure closely followed that of our previous work (Davis et al. 2021; Henderson and Campbell 2023). Following EEG set-up (a process involving fitting a fabric cap, gelling and placing electrodes, and troubleshooting connection issues), participants viewed the movie with the instructions that they should watch it just like they might at home. Immediately following the movie, participants were given a cued recall task in which they watched short video clips from the movie (either from the middle or end of scenes) and were asked to report what happened immediately following the clip. Cues were presented in a set chronological order, and all participants saw the same cues. Participants then performed a free recall task in which they were asked to describe the movie they watched in as much detail as possible. They had unlimited time to respond in both memory tasks and their responses were audio recorded and later transcribed.

Following the memory tasks, participants completed a questionnaire evaluating whether they were aware that their memory for the movie would be tested and whether they believed this knowledge influenced what they attended to in the movie. They then completed the MoCA (Nasreddine et al. 2005), Shipley vocabulary test (Shipley 1940), the Image of Aging Scale (Levy et al. 2004), and a demographic questionnaire.

### Scoring

#### Cued recall

Responses were transcribed and then coded for accuracy (binary correct or incorrect) as well as the type of errors that were made. Responses were considered to be correct when they described anything that occurred in the target scene (the same scene for within-event cues and the next scene for between-event cues) which was not evident from the cue itself. The scoring was relatively liberal such that the inclusion of incorrect details within responses otherwise describing the gist of the correct scene was marked as correct; however, these errors were also recorded. Errors were coded into several categories: incorrect information (describing something that did not occur), earlier scenes (describing something that occurred earlier), next scenes (describing the scene following the target), and far errors (describing something that occurred two or more scenes after the target). The data were scored by two raters with 25% overlap (*n* = 12), and the coding for accuracy was identical across both raters. Raters were blind to participant age group. However, as scoring which scene was being described required knowledge about scene content and perceived boundaries, raters were aware of the cue categories (within vs between).

#### Free recall

Responses were transcribed and coded using a modified version of the Autobiographical Interview (Levine et al. 2002) adapted for naturalistic stimuli by Fenerci et al. (2024). This method involves first segmenting the transcript into distinct details (ie stand-alone units of information). Details are then coded into several possible categories describing their content. Details which accurately describe the stimulus are categorized as internal details. Internal details are subdivided into three categories: event details, conceptual details, and perceptual details. Event details describe the actions that further the story (eg “he was handed a drink”). Conceptual details are those that can be reasonably inferred from the stimulus but were not explicitly shown (eg “she was worried”). Perceptual details are those that describe the context in which the events occur and sensory details therein (eg “there was a suitcase on the bed”). Details that were non-episodic were considered external details (as in Levine et al. 2002). These include any detail that reflects semantic information (eg general knowledge or facts), repetitions of previously reported internal details, metacognitive statements (eg “I don’t remember this part”), or editorial statements. Finally, details that were described as though they occurred in the movie but did not were coded as incorrect details. Two raters scored the data with overlap over eight files (four from younger adults and four from older adults). Interrater reliability was excellent for the overall number of details (ICC = 0.99) and across all categories of details (all ICCs > 0.80) reflecting similarity both in the segmentation and categorization of details. These memory performance data from the free recall have been previously analyzed by Fenerci et al. (2024).

### Analysis

#### Preprocessing

EEG data were preprocessed using PyLossless (Huberty et al. 2024), which is a Python-based implementation of the EEG Integrated Lossless Platform developed by Desjardins et al. (2021). The data were common average re-referenced, noisy sensors and time periods with outlying voltage variance were automatically flagged, the data were notch filtered at 60 Hz, and uncorrelated sensors and time points as well as bridged sensors were flagged. All pipeline parameters can be found at the OSF link. A high-pass filter was not applied as we wanted to preserve the large-scale shifts in activation that may reflect perceived events. ICA was run to categorize signals as neural, muscle artifacts, eye blinks, heartbeat, line noise, channel noise, and other non-cortical signals using ICLabel in Python (Li et al. 2022). ICs identified with at least 30% confidence as a category other than brain or other signal were subtracted from the data. This resulted in the removal of data from an average of 20.2 ICs (*SD* = 8.22) of an average total of 109 ICs (*SD* = 6.94). These pipeline decisions were implemented to remove the identified non-neural data and noise, and the resulting data were projected back to the scalp channels. Flagged channels were then interpolated following the spherical spline method from this cleaned version of the data. Flagged time points were not removed as the GSBS approach requires continuous data; however, very few time points were flagged on average, suggesting that any remaining artifacts did not have a significant impact on the results (*M* = 1.55 flags per subject with an average duration of 2.2 s). Pipeline decisions were visually evaluated for suitability to the data in a subsample of five older and five younger adults.

#### Neural state segmentation

##### Greedy State Boundary Search

**Fig. 1:**
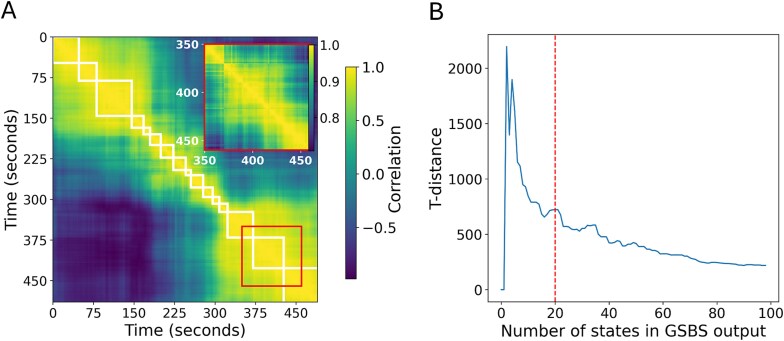
Neural pattern similarity with GSBS outputs for one example participant. (A) Time by time pattern similarity overlaid with GSBS boundary output for an example participant (older adult). Comparing the full matrix to the inset matrix reveals the occurrence of temporally nested states. (B) *t*-Distance plot from GSBS output for the same participant showing the *t*-distance metric for each iteration of the GSBS algorithm. The optimal selected number of states we identified is marked with a dotted line.

Neural state boundaries were identified within each individual’s EEG data using the GSBS algorithm (Geerligs et al. 2021, 2022). This algorithm iteratively searches for state boundary locations that maximize the similarity in the pattern of activity within states (see Fig. 1 for an example participant). The input to the algorithm is a time by electrode matrix. For each iteration of the algorithm, an existing state is split into two or three new states by placing one or two new boundaries. This is done by performing a search across all possible temporal locations and calculating the average correspondence of the underlying neural data at each timepoint with the mean activity pattern of the proposed states (state template). The optimal boundary location(s) is selected as the location(s) which maximizes the similarity of each timepoint in the data with the state template (Geerligs et al. 2021, 2022). On every iteration, previously placed boundaries may be fine-tuned by shifting them one sample in either direction to maximize the within-state correlation given the placement of each new boundary.

The maximum possible number of states was set to the length of the recording in seconds (489 s). This value was selected to reduce computation time (versus using one half of the total number of time points), as we found in preliminary tests that the fit statistic (*t*-distance) plateaued after ~100 boundaries were placed. One of the benefits of the GSBS algorithm is that it requires no input assumptions about the number of states in the data (Geerligs et al. 2021). The optimal number of states can be determined using the *t*-distance metric which quantifies the distance between the distribution of the within-state correlations and the distribution of correlations between consecutive states.

In preliminary analyses, we observed strong patterns of anti-correlations over very long time scales (>100 s). This meant that the *t*-distance values were extremely high for solutions with very few boundaries (ie a single placed boundary was optimal). Here, we were interested in studying neural states that occurred at a temporal grain similar to or more fine grained than event segmentation. Therefore, we set the minimum number of states to 15 and identified the first peak in *t*-distance beyond this minimum as the optimal fit for that individual. This minimum value was selected to fall approximately in line with the scale of behaviorally identified event boundaries in the movie (previous work identified 19 events in this movie; Ben-Yakov and Henson 2018). Figure 1B shows an example *t*-distance plot showing the extreme peak observed for the two and four state solutions and the smaller peak reflecting the fit obtained by limiting the minimum number of states.

Since previous work shows that frontal networks best reflect large-scale, behaviorally relevant event states (Baldassano et al. 2017; Geerligs et al. 2022), we applied the algorithm to a large number of frontal electrodes (32 electrodes encompassing Fz, F3, F4, F7, F8, and Fpz of the 10–20 system; see Supplementary Fig. 2). Some work has also linked activity in the lateral parietal and temporal regions to event segmentation (though notably, following the posterior-to-anterior gradient of neural segmentation, neural states in these regions are typically observed on a finer time scale; Sava-Segal et al. 2023), so we also replicated our analyses with a large set of temporal and parietal electrodes (see “Parietal GSBS” in Supplementary Materials). To reduce the computational complexity, the data were down-sampled before being submitted to the GSBS procedure. This was accomplished by averaging the 512-Hz recordings over every 100 samples, resulting in approximately one sample every 200 ms. Similar sampling rates and procedures have been used previously in the neural state segmentation of EEG data (Silva et al. 2019).

##### Quantifying between-subject state alignment

We assessed GSBS-output similarity between individuals by computing the relative correlation between an individual’s boundary locations and the group average locations (see Fig. 2.2). Similar to methods used to assess overlap between behavioral event segmentation performance (Sasmita and Swallow 2022), the individual GSBS outputs (ie boundary locations) were convolved with a two-sample *SD* (~400 ms) Gaussian distribution over each boundary to account for minor variability in boundary identification. For each individual, the average location of all other subjects’ boundary output was calculated in a leave-one-out manner and then correlated to the individual’s convolved output timeline. The null distribution of the correlation between the individual and the group (collapsed across age groups) was then computed by shuffling the order of the individual’s states (while maintaining their duration) 1,000 times, applying the same Gaussian over each shuffled boundary, and correlating it to the group average. To correct for individual differences in the maximum possible overlap due to the number of identified boundaries within each person, both the null and test correlations were adjusted according to the maximum possible correlation between the individual and group average boundaries. This was done by placing boundaries over the *n* highest peaks in the group average timeseries where *n* equals the number of states in the individual’s GSBS output. Adjusted match to the group was computed as (correlation to group/maximum correlation to group) − (mean of null distribution/maximum correlation to group) (see Fig. 2.3).

**Fig. 2:**
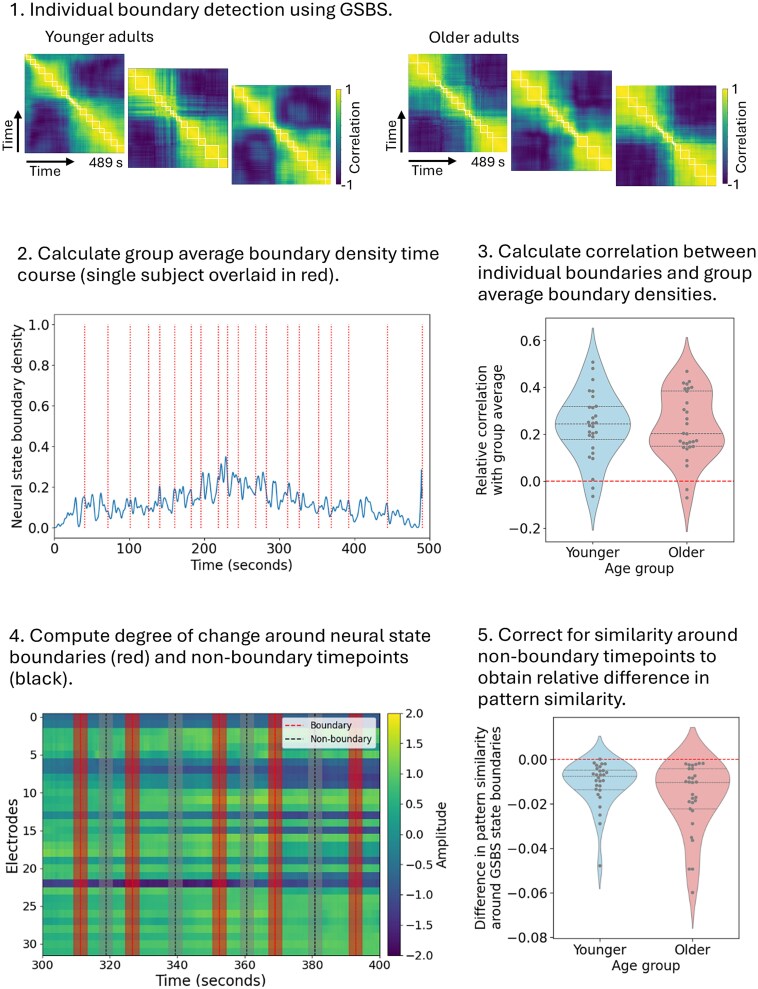
Overview of EEG analyses. (1) Neural state boundaries determined separately for each individual. (2) Average state boundary location density determined for the group. Vertical lines represent the neural state changes from a random single participant. (3) Correlation between each individual and the group-level boundary density (in a leave-one-out manner) computed and adjusted for the maximum possible correlation (based on number of boundaries) in each individual. More positive values reflect greater alignment with the group. (4) Degree of change around state boundaries and non-boundaries computed for each individual by finding the average correlation for all time points in the 2 s before compared to 2 s after boundary and non-boundary timepoints. (5) Relative change around boundaries computed by subtracting the median similarity around non-boundary timepoints from the change around state boundaries. More negative values reflect a larger shift around boundaries compared to other time points.

##### GSBS alignment with behaviorally defined boundaries

The location of boundaries identified with group-level GSBS applied to fMRI data have previously been shown to align with behaviorally defined event boundaries (Geerligs et al. 2021, 2022). Thus, we investigated the average alignment of the individual-level GSBS output used here with boundaries obtained from an event segmentation task. The location of perceived events was determined using a separate sample of older (*n* = 30; 65 to 80 years) and younger participants (*n* = 36; 18 to 30 years). Participants viewed the movie and were asked to press a key when they thought one event had ended and another had begun. In line with previous work (Ben-Yakov and Henson 2018), we subtracted 0.9 s from each button press to account for response time, and then for each timepoint, computed the proportion of subjects that pressed within a 2-s window. The density of the presses was calculated across all participants, and in the younger and older groups alone. The density of responses was then used to quantify the degree of alignment between the behaviorally defined boundaries and each individual’s neural state output. We assessed this alignment with a similar method as the between-subject alignment. For each individual, the location of their GSBS-identified boundaries was convolved with a 400-ms SD Gaussian distribution over each boundary and correlated with the density of the behavioral responses both in the entire group and in their age group alone. The null distribution of the correlation between the individual and the behavioral boundaries was then computed by shuffling the order of the individual’s states (while maintaining their duration) 1,000 times, applying the same Gaussian over each shuffled boundary, and correlating it to the behavioral densities time course. As with previous analyses, to correct for individual differences in the maximum possible overlap due to the number of identified boundaries within each person, both the null and test correlations were adjusted according to the maximum possible correlation between the individual and behavioral boundary data. This was done by placing boundaries over the *n* highest peaks in the behavioral density timeseries where *n* equals the number of states in the individual’s GSBS output. Adjusted match to the behavioral data was computed as (correlation to group/maximum correlation to group) − (mean of null distribution/maximum correlation to group). We tested this effect both for the overall group and with only age-matched group behavioral data.

##### Pattern similarity around state boundaries

The relative distinctiveness of state boundaries was computed by calculating the average correlation between the 2 s around each boundary adjusted for the average correlation at non-boundary timepoints in the center of each state (see Fig. 2.4). The GSBS algorithm outputs a similar measure of boundary strength, reflecting how large the differences between overall patterns of activity are between subsequent neural states (Geerligs et al. 2021, 2022), but we aimed to look at the degree of change immediately around state boundaries (not across the entire state). Thus, for every state boundary, we calculated between-state correlation values. This was done by first computing the correlation between each timepoint before the boundary and each timepoint after the boundary. Next, these correlations were averaged across all timepoint pairs separately for each boundary. Finally, we took the median of these between-state correlation values across all boundaries to account for the skewness of the distribution. Since these between-state correlation values could partly reflect individual differences in the degree of correlation between successive timepoints overall (irrespective of the location of neural state boundaries), we corrected by subtracting within-state correlation values. These were based on the correlation in the 2 s around times from the middle of neural states, computed using the same procedure as above (see Fig. 2.4). Thus, negative values indicate that the time around boundaries was more distinct than times taken from the middle of the states.

##### Neural state correlations with memory

We assessed the relationship between memory for the movie and our two GSBS measures of interest (between-subject state alignment and pattern similarity around state boundaries) using two separate regression analyses. Our outcome measure of interest for this analysis was the proportion of internal details (ie the number of internal details that accurately described some aspect of the movie divided by the total number of details). This proportional measure is commonly used in the aging and autobiographical memory literature as it helps correct for individual differences in narrative style and overall verbosity (eg Levine et al. 2002; Cole et al. 2013; Peters and Sheldon 2020), and has improved trait estimation of memory performance (Lockrow et al. 2024). We did not assess the relationship between our neural measures and performance on the cued recall task because the cues for this task were based on scene changes that did not necessarily align with all the individual state changes. Both regression models included age group and the interaction between age and the neural measure of interest as predictors. Coefficient estimates were based on the median of the bootstrapped distribution obtained from 5,000 model replications.

## Results

### Behavioral

Previous work has shown that within-event cued recall is more accurate than between-event cued recall reflecting the stronger associations formed between details within the same event. Cued recall accuracy was assessed using a 2 Age (between-subjects; old, young) by 2 Cue Type (within-subjects; within-event, between-event) ANOVA. This revealed the expected main effect of cue type, such that memory was better following within-event (*M* = 0.75, *SD* = 0.20) than between-event (*M* = 0.62, *SD* = 0.23) cues, *F*(1, 52) = 22.3, *P* < 0.001, η^2^*_p_* = 0.300. The main effect of age was not significant, *F*(1, 52) = 3.16, *P* = 0.081, η^2^*_p_* = 0.057, nor was the two-way interaction between age group and cue type, *F*(1, 52) = 1.57, *P* = 0.216, η^2^*_p_* = 0.029. Thus, younger and older adults did not significantly differ in their cued recall performance and within-event recall was significantly better than between-event recall. See Table 1 for means separated by age group.

**Table 1:** Mean cued and free recall performance separated by age group.

	Younger adults (*n* = 28) *M* (*SD*)	Older adults (*n* = 26) *M* (*SD*)	*t* (*P*)
Cued recall			
Within-event cue	0.779 (0.160)	0.727 (0.236)	0.949 (0.347)
Between-event cue	0.677 (0.198)	0.551 (0.248)	2.06 (0.045)^*^
Free recall			
Event	54.7 (25.1)	57.0 (18.9)	0.381 (0.704)
Perceptual	10.3 (5.83)	12.9 (9.37)	1.25 (0.217)
Conceptual	5.50 (4.97)	10.7 (7.55)	3.01 (0.004)^*^^*^
External	5.00 (4.55)	10.3 (10.5)	2.47 (0.017)^*^
Incorrect	4.32 (2.93)	5.19 (3.32)	1.02 (0.311)
Proportion internal	0.888 (0.049)	0.853 (0.063)	2.28 (0.026)^*^

Statistics reflect the independent samples *t*-test comparing older and younger adults. ^*^P < 0.05, ^*^^*^P < 0.01.

Our primary measure of interest from the free recall task was the proportion of internal details recalled. An independent samples *t*-test revealed that older adults recalled a significantly lower proportion of internal details on average, *t*(52) = 2.28, *P* = 0.026, *d* = 0.622. However, as can be seen in Table 1, younger and older adults were quite similar in the number of details they recalled overall, with older adults recalling significantly more conceptual and external details (for a detailed discussion of these recall data, see Fenerci et al. 2024). Thus, the age differences observed here are quite subtle and may partly reflect age differences in narrative style or views about what is most important to recall.

### Electroencephalography

#### Individual neural state detection

We first evaluated the relative similarity across individuals in their state boundary placements. To this end, the relative correlation of each individual’s GSBS output to the group average GSBS output was calculated and adjusted according to the maximum possible match (depending on both the group mean and the number of boundaries in the individual’s GSBS output). This was further adjusted using the null distribution for each individual (computed by shuffling the order of the states 1,000 times) such that the adjusted match value reflects the relative similarity to the group with a value >0 indicating that the correlation was higher than the mean of the individual’s null distribution and a match of 1 reflecting the maximum possible correlation.

When we identified the number of states in each individual, we observed that there were temporally nested neural states that occurred at different timescales (see Fig. 1 for an illustration). The slowest timescales dominate the *t*-distance curve, but do not occur at the temporal scale of event segmentation we are interested in. That is why the number of states identified in each individual was set to a minimum of 15, but was allowed to vary after this point, such that the first peak in *t*-distance (defined as the first point at which the *t*-distance of neighboring model fits in both directions were smaller) after 15 states was used as the optimal fit for each participant. The number of states identified in this way did not differ between younger (*M* = 17.6, *SD* = 2.93) and older (*M* = 18.3, *SD* = 3.41) adults, *t*(52) = 815, *P* = 0.419, *d* = 0.222.

#### Between-subject state alignment

The association between individual and group GSBS boundaries (averaged across all participants) was significantly above chance in both younger (*M* = 0.237, *SD* = 0.141), *t*(27) = 8.90, *P* < 0.001, *d* = 1.68, and older adults (*M* = 0.223, *SD* = 0.144), *t*(25) = 7.90, *P* < 0.001, *d* = 1.55 (see Fig. 2.3 for the distribution of adjusted match statistics within each age group). An independent samples *t*-test revealed that there was no age difference in the average correlation to the group, *t*(53) = −.368, *P* = 0.714, *d* = −.100. Thus, GSBS identified neural state changes that significantly aligned across participants, and somewhat surprisingly, this alignment did not differ with age. We replicate this effect for parietal sites (see Supplementary Materials).

#### Individual GSBS alignment with behaviorally defined boundaries

The association between behaviorally defined events and individual GSBS neural state boundary locations was not significantly greater than the similarity to shuffled boundary locations in the overall sample (*M* = −.010, *SD* = 0.129), *t*(53) = −.575, *P* = 0.568, *d* = −.078, nor in younger (*M* = 0.006, *SD* = 0.126), *t*(27) = 0.251, *P* = 0.804, *d* = 0.047, and older adults separately (*M* = −.051, *SD* = 0.239), *t*(25) = −1.08, *P* = 0.291, *d* = −.212, when compared to behavioral event boundary densities obtained from a separate group of age-matched participants. An independent samples *t*-test revealed no age difference in the average association with behaviorally identified boundaries (averaged across all participants), *t*(52) = 1.18, *P* = 0.244, *d* = 0.321. We replicate these effects for parietal sites (see Supplementary Materials). Thus, neural states identified using GSBS did not significantly align with the location of event boundaries identified by a separate group of participants engaging in intentional boundary identification.

Since this lack of alignment with behavioral events was slightly unexpected, we also assessed pattern similarity within *behaviorally derived* events in a manner similar to that of Silva et al. (2019; see Supplementary Material). We found that at the level of whole events, pattern similarity was higher within events than would be expected by chance. However, we did not observe a significant shift in the pattern of neural activity in the 2 s immediately surrounding behaviorally derived event boundaries (like that observed for GSBS-defined neural state transitions; see “Pattern Similarity Around State Boundaries” below). Together, these results are consistent with the notion that behaviorally derived events represent meaningful ongoing states of the film content that have distinct neural representations on average, but may not align precisely enough across participants to be captured by a change in neural activity right at event boundaries, which may require greater temporal precision.

#### Pattern similarity around state boundaries

The difference in pattern similarity around neural state boundaries (compared to non-boundary timepoints) was significantly above chance in both younger, *t*(27) = 5.57, *P* < 0.001, *d* = −1.05, and older adults, *t*(25) = 7.90, *P* < 0.001, *d* = −1.55 (see Fig. 2.5 for the distribution of pattern similarity around state boundaries for each age group). Thus, across groups, there was a larger change in the pattern of activation in the 2 s before and after an identified boundary than at random time points in the movie. This would be expected as the GSBS algorithm aims to maximize differences between subsequent states. An independent samples *t*-test revealed that there was no difference between younger (*M* = −.011, *SD* = 0.010) and older (*M* = −.017, *SD* = 0.015) adults, *t*(52) = −1.72, *P* = 0.091, *d* = −.469. Thus, contrary to our prediction, older adults did not show less distinct shifts in activity at these state change boundaries. We replicate these effects for parietal sites (see Supplementary Materials).

#### Neural state correlations with memory

##### Between-subject state alignment

**Fig. 3:**
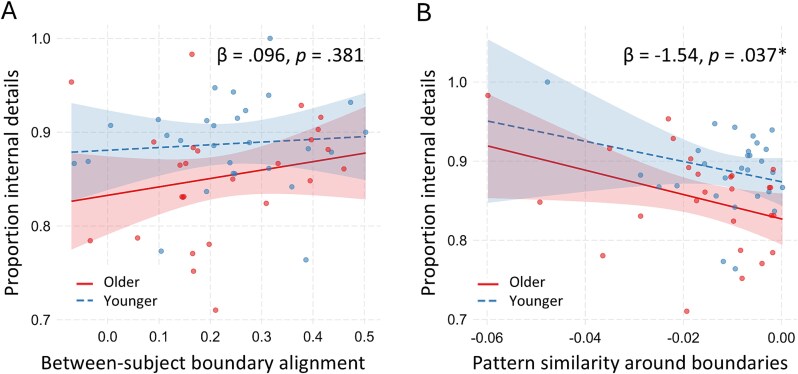
Memory performance predicted by between-subject state alignment and pattern similarity around state boundaries. Proportion of internal details calculated as the number of internal details (reflecting some aspect of the movie) divided by the total number of details provided during free recall. (A) Between-subject boundary alignment calculated as similarity to all other individuals’ neural state boundary placement adjusted for the maximum possible alignment for each individual. (B) Pattern similarity in the 2 s surrounding neural state boundaries adjusted for individual’s pattern similarity in the center of each neural state. Shaded areas represent 95% CI.

We next evaluated whether individual differences in alignment to the whole group would predict memory performance, with the expectation that greater alignment with the group should relate to better memory (eg Hasson et al. 2004; Davis et al. 2021). A linear regression was run predicting the proportion of internal details recalled from the adjusted correlation between each individual and their group, the individuals’ age group, and the interaction between these two factors. The model did not significantly improve fit over the null model, *F*(3, 50) = 2.20, *P* = 0.099. Within this model, age group was not a significant predictor of memory performance (β = 0.048, *P* = 0.111), an individual’s correlation with the group did not predict memory performance (β = 0.096, *P* = 0.381), and the two-way interaction was not significant (β = −.066, *P* = 0.629). This suggests that alignment with the group did not predict memory performance (see Fig. 3A for regression lines for each age group and individual data points; see Supplementary Material for a similar result with parietal sites).

##### Pattern similarity around state boundaries

Finally, we assessed whether pattern similarity around state boundaries would predict memory performance, with the expectation that a greater change in the pattern of activity at state boundaries (ie more distinct boundaries) should relate to better memory for the movie. A linear regression was run predicting the proportion of internal details recalled from the relative neural pattern similarity in the 2 s surrounding neural state boundaries (adjusted for the similarity in the center of neural states), age group, and the interaction. The model significantly improved fit over the null, *F*(3, 50) = 3.93, *P* = 0.014. Within the model, age group significantly predicted memory performance (β = 0.050, *P* = 0.034), such that younger adults performed better on average. The relative similarity around neural state boundaries also predicted memory performance (β = −1.54, *P* = 0.037), such that lower correlations around GSBS boundaries (ie more distinct boundaries) predicted higher memory performance. The interaction between age group and pattern similarity around boundaries was not significant (β = 0.509, *P* = 0.973). We see a similar pattern of results for parietal sites (see Supplementary Materials). Thus, memory performance was higher for individuals with more distinct boundaries as reflected by lower correlation of neural activity in the 2 s before and after a state boundary, and this effect did not differ according to age group (see Fig. 3B for regression lines for each age group and individual data points).

## Discussion

In the current study, we used neural state segmentation to determine the unique contributions of normative state segmentation (ie correspondence of neural states across people) and relative boundary strength to age differences in memory for the movie. We employed a well-established method of neural state segmentation (Geerligs et al. 2021, 2022) in a novel way to derive boundary locations for each subject. We found that the state segmentation method was effective in identifying neural state boundaries that were shared between subjects, though they did not align with perceived event boundaries identified by a separate sample of younger and older adults. Between-subject alignment of neural state boundaries was not a significant predictor of memory for the movie. However, the relative difference in neural pattern similarity around neural state boundaries did predict memory performance such that more distinct boundaries predicted a higher proportion of internal details recalled. Similar effects were observed for frontal and parietal sites. Older and younger adults did not differ in their correspondence to the group boundary locations, nor in boundary distinctiveness. Furthermore, the relationship between boundary distinctiveness and memory performance was not moderated by age, suggesting that more distinct neural state transitions relate to better memory regardless of age.

Behaviorally, younger adults performed better than older adults in the free recall task as measured using the proportion of internal details (ie the number of details accurately describing some aspect of the movie divided by the total number of details produced). In contrast, there were no significant age differences in cued recall, nor was there an interaction between age group and cue type. Based on our previous work showing that specifically low-performing older adults show a relatively smaller difference between within- and between-event cues (Henderson and Campbell 2023), we expected older adults to show at least a trend toward a smaller difference between cue types than younger adults. However, this was not the case and if anything, older adults show a numerically larger difference between cue types (*M*_diff young_ = 0.102, *M*_diff old_ = 0.176). This may be because the current sample of older adults was quite high performing on average. For instance, compared to older adults in the study by Henderson and Campbell (2023), older adults in the current sample had numerically more internal and fewer external details in their free recall. Thus, the lack of age difference in cued recall is not surprising, and this may also help explain the lack of age difference at the neural level.

Previous work has supported the use of GSBS (Mariola et al. 2022) and neural state segmentation algorithms in general (Silva et al. 2019) for electrophysiological data, though typically, with group-averaged data. Some recent evidence suggests that stable individual neural state segmentation may be possible with fMRI (Betzel et al. 2022; Sava-Segal et al. 2023). Here, we extend these findings to narrative stimuli in older and younger adults. Based on previous observations of between-subject overlap in both perceived event boundaries (Zacks et al. 2009; Kurby et al. 2014) and neural states (Sava-Segal et al. 2023), we expected to see correspondence in neural state boundaries across participants that exceeded the overlap expected by chance. As predicted, we show that boundaries overlapped significantly across individuals. Although these boundaries significantly overlap, it is worth noting that there is significant idiosyncrasy in boundary location as evidenced by the numerical value of the overlap scores (*M*_younger_ = 0.237, *M*_older_ = 0.223) which can theoretically reach a maximum of one (wherein each of *n* boundaries would align with the *n* timepoints of highest density observed in others). This idiosyncrasy may reflect individual differences in boundary perception that have been shown to be internally consistent within individuals (Betzel et al. 2022) and reflect differences in the appraisals of stimuli and variability in the memories thereof (Sava-Segal et al. 2023). This reinforces the notion that using individual boundary detection may be critical in identifying the neural underpinnings of event segmentation.

Previous work has found significant alignment between event boundaries obtained from explicit event segmentation tasks and the neural state boundaries identified by GSBS in fMRI (Geerligs et al. 2021; Geerligs et al. 2022). We did not find this alignment in the current study, but our current approach differs in both imaging modality (EEG vs fMRI) and its application to individual subject versus group-averaged data. Previous studies looking at neural state segmentation using EEG did not test this question directly (ie measuring the alignment between behavioral and neural boundaries in narrative stimuli; Mariola et al. 2022; Silva et al. 2019). While Mariola et al. (2022) did show alignment between behavioral and GSBS-derived boundaries, they used much shorter video clips (ranging in length from 10 to 64 s) from several content categories (eg city, countryside) that did not contain narrative information. Furthermore, participants were instructed to focus on perceptual and semantic information in their behavioral segmentation, resulting in much more finely grained segmentation that may more closely align with low-level perceptual shifts. In contrast, Silva et al. (2019) used behaviorally derived event segmentation models to show that patterns of neural activation during encoding were more similar within an event compared to a null distribution of shuffled events (an effect we replicate in the Supplementary Material), but did not use neural state segmentation methods to determine boundary locations during encoding and, thus, did not test temporal alignment of these boundaries.

The lack of alignment between behavioral boundaries and neural state changes observed here may be due the mixed nature of the EEG signal. fMRI work has shown that different brain regions can show neural state boundaries at different moments and different timescales and that some, but not all, boundaries in time and space align with perceived event boundaries (Baldassano et al. 2017; Geerligs et al. 2022). Given the mixed spatial origin of the signal recorded from scalp-level EEG, the different neural state boundaries over time in a single participant could be driven by different neural sources, which could be a cause for this reduced correspondence to perceived event boundaries. In line with this argument, we observed similar effects for both frontal and parietal sites. Furthermore, it should be noted that previous fMRI work showing significant alignment between neural state changes and perceived event boundaries focused on stimulus-induced neural states by applying GSBS to group-averaged data. In the current single-subject approach, though the alignment in neural states between individuals points to states that are at least partially driven by the stimuli, they could also be influenced by changes in the participants’ internal states (eg mind wandering, recalling related events from one’s own past). This could also contribute to the reduced alignment between neural states and behaviorally identified events.

The lack of alignment could also simply reflect real individual differences in event segmentation. Behavioral event segmentation work has shown that in addition to points of agreement shared between individuals, there is substantial variability in when individuals segment naturalistic stimuli (Zacks et al. 2009; Sargent et al. 2013; Sasmita and Swallow 2022; Sava-Segal et al. 2023). There are many potential sources of this idiosyncrasy. Perhaps the most well studied is the notion that it could reflect individual differences in how information is integrated with pre-existing schemas or event knowledge. This is consistent with work showing that individuals with varying levels of knowledge about a topic tend to segment and perceive events differently (Van Kesteren et al. 2012; Bläsing 2015; Smith et al. 2020; Newberry et al. 2021; Pförtner and Hristova 2024), potentially changing the duration or the location of perceived boundaries/neural states. Beyond schematic representations of events, idiosyncrasy in event segmentation may also reflect differences in what aspects of events are most salient and, as a result, in what an individual attends to (Thompson et al. 2018; Samur et al. 2021) and later remembers (Geissmann et al. 2023; Haro et al. 2024). For instance, while emotional information tends to increase group-level neural synchrony (Song et al. 2021), there are individual differences in emotional fluency which may shift how people understand events (Vega 1996; Samur et al. 2021; Schmidt et al. 2023) and, as a result, how they represent them neurally (Bacha-Trams et al. 2018). Indeed, instructions to attend to different aspects of a narrative have been shown to influence how it is segmented (Bailey et al. 2017), highlighting the importance of top-down attention in event model processing. Ultimately, when watching a film, or indeed, when engaging with our world, individual differences in our knowledge, personality, and fluid cognitive abilities shape how we attend to and process complex, multifaceted stimuli. These potential contributors to individual neural state segmentation illustrate the richness of applying more individualistic models to segment continuous neural data. Taking an individual approach to neural segmentation may inform our understanding of both stimulus-driven and internally driven factors that contribute to the way we parse our ongoing experiences (for review see, Finn et al. 2020). Thus, more work that takes an individualized approach to attention, neural state segmentation, and event boundary perception may be required to better understand these idiosyncrasies.

In this EEG application of the GSBS algorithm we observed nested states over relatively long time scales (up to several hundred seconds). This observation is consistent with the partially nested nature of neural states that have been described in fMRI data across different cortical areas (Baldassano et al. 2017; Geerligs et al. 2022). They also reinforce the notion that as a result of the mixed spatial original of the signal recorded from scalp-level EEG, the observed neural states reflect a combination of internally and externally driven states with various sources in the brain. In this work, we specifically focused in one temporal scale of state segmentation, but studying states at different temporal scales may be another rich source of information to better understand how the brain naturally segments experience into meaningful units.

This temporally nested nature of states in EEG data did provide a challenge in identifying an optimal number of states using the *t*-distance metric developed previously for fMRI (Geerligs et al. 2021, 2022). As illustrated in Fig. 1, it meant that simpler solutions with fewer states were heavily favored using the *t*-distance metric, with *t*-distance values typically favoring a two-state solution. We overcame this issue by setting a minimum number of possible states in the range of the expected number of perceived states based on behavioral segmentation of the movie and selecting the next peak in *t*-distance. While boundaries are placed in order of strength (ie more distinct neural state boundaries tend to be placed first), and thus, setting a minimum number of states would not affect these strongest boundaries, setting a minimum does limit our ability to investigate nested neural states or the number/length of neural states across individuals. Future work may seek to refine this metric for use in EEG data, particularly when longer timescales are the focus of investigation.

While previous behavioral studies have shown that more idiosyncratic event segmentation relates to poorer subsequent memory (Zacks et al. 2006; Bailey et al. 2013), we found that individual differences in neural state agreement did not predict memory for the movie (though as can be seen in Fig. 3A, the relationship was in the expected direction). Notably, we observe similar effects in our parietal application of GSBS (see Supplementary Material) in regions that have been associated with somewhat higher interindividual boundary agreement compared to relatively more idiosyncratic frontal regions (Sava-Segal et al. 2023). In studies that use explicit segmentation tasks, the relationship between segmentation agreement and memory may, at least partially, reflect other cognitive processes that are shared by both tasks, such as the ability to maintain attention (Ryan and Rogers 2021; Madore and Wagner 2022) or the ability to divide attention between the primary task of watching the movie and the secondary task of indicating when a boundary has occurred (Riby et al. 2004; Ataseven et al. 2023). This may help to explain the mixed findings in the event segmentation literature surrounding older adults’ ability to segment events. Though older adults often show more idiosyncratic event segmentation during intentional or explicit event segmentation tasks (Zacks et al. 2006; Kurby and Zacks 2011; Bailey et al. 2013), implicit measures tend not to show age differences (Morrow et al. 1997; Radvansky and Curiel 1998; Kurby and Zacks 2018; Davis et al. 2021; Smith et al. 2023). It is possible that explicit event segmentation tasks are particularly difficult for older adults, requiring effortful attention to a process that normally occurs automatically. The current findings are more consistent with the notion that event perception is partly determined by knowledge and event schemas that can differ across individuals and affect when a boundary is perceived (Bläsing 2015; Smith et al. 2020; Newberry et al. 2021). Individual differences in knowledge also likely contribute to differences in the type of information recalled (Castel et al. 2007; Hemmer and Steyvers 2009; Umanath and Marsh 2014; Fenerci et al. 2024).

Memory performance was found to be predicted by the change in neural activity around state boundaries adjusted for pattern similarity in the middle of neural states. This effect did not differ across age groups and was replicated across frontal and parietal electrode clusters (see Supplementary Materials), suggesting that more distinct boundaries support successful memory encoding regardless of age. This is consistent with work showing that more distinct patterns of activation in the hippocampus and medial prefrontal cortex during movie watching are associated with subsequently remembered (vs forgotten) events (Liu et al. 2022). Furthermore, it is consistent with our previous behavioral work suggesting that older adults who encode more distinct events (and are better able to update working memory) show better memory for the movie overall (Henderson and Campbell 2023). Event memory seems to benefit from the encoding of distinct units (Ezzyat and Davachi 2011; Davis and Campbell 2022), possibly because these aid in the reinstatement of more distinct contexts at retrieval (Lohnas et al. 2023). Blurring across event boundaries may thus result in disorganized, indistinct event representations that impair recall performance. More distinct neural states predicting better memory performance is also in line with work arguing that age differences in the ability to suppress irrelevant information, including previously attended information that is no longer relevant, contribute to age differences in memory (Hasher and Zacks 1988; Campbell et al. 2020; Weeks et al. 2020). While further work is required to determine if individual differences in inhibitory control relate to the distinctiveness of neural state changes, the current work suggests that greater neural change at these boundaries is good for memory.

## Conclusion

We employed GSBS to identify neural state transitions in older and younger adults’ EEG data during movie watching. We found that boundary location significantly overlapped between individuals and their peers. However, the degree of overlap did not predict memory performance, nor did it vary with age, in line with previous work showing minimal difference in event segmentation with age when measured implicitly. Memory for the movie was predicted by the distinctiveness of neural state boundaries, with more distinct boundaries relating to a greater proportion of internal details being recalled. Although we cannot speak to the precise mechanism through which boundary distinctiveness improves memory, this points to the importance of how event boundaries are processed rather than when they are perceived for successful encoding.

## Supplementary Material

MVEEG_Supplementary_Materials_Resubmission

## Data Availability

Behavioral and EEG data and code supporting the findings of this study are at available at https://osf.io/vc8s3/.
