# Supplementary material for: Neural state changes during movie watching relate to episodic memory in younger and older adults
Source: Cereb Cortex. 2025 May 19;35(5):bhaf114. doi: 10.1093/cercor/bhaf114 (PMC12086427; doi:10.1093/cercor/bhaf114)
Supplement: MVEEG_Supplementary_Materials_Resubmission [file mveeg_supplementary_materials_resubmission.docx]

**Supplementary Materials**

**Alignment between GSBS neural states and behavioural boundaries**

Behaviourally-derived event boundaries from a separate group of individuals did not align significantly with neural state boundaries (see Supplementary Figure 1). Notably, the behavioural data has larger peaks in agreement than neural state boundaries. This may be partially due to task instructions during the behavioural segmentation task directing participants to pay attention to moments of narrative and contextual shifts. These task demands may work to enhance intersubject agreement by directing attention toward stimulus content, especially observable shifts in sensory and narrative features of the stimuli. Conversely, the absence of a task may allow for greater intersubject idiosyncrasy as individuals attend to different features of the stimulus or internally generated thoughts (e.g. integrating content with past experiences, opinions about aspects of the narrative, task-unrelated thoughts, etc.).


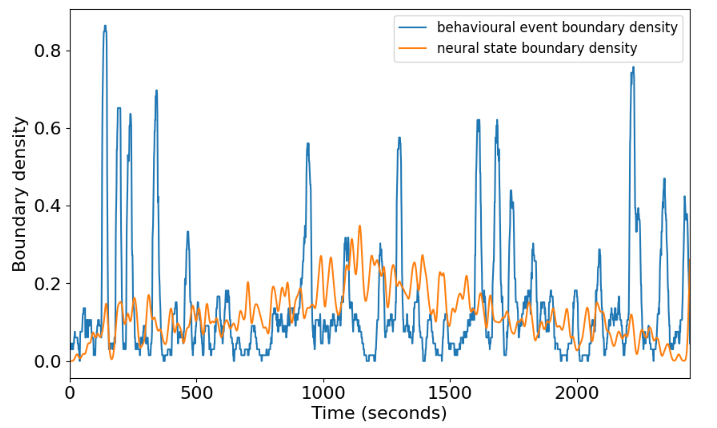


*Supplementary Figure 1*. Alignment between group boundary density obtained from GSBS neural state segmentation at frontal sites (*n* = 54) and group boundary density obtained from the separate sample who performed a behavioural segmentation task (*n* = 66). All data collapsed across age groups.

**Behavioural event model pattern similarity**

**Whole event.** We aimed to replicate previous work which has shown that pattern similarity is higher within behaviourally-defined events than the null distribution of shuffled events (Silva et al., 2019). To this end, we calculated the average correlation of activity across each timepoint (200ms/sample) and computed the median correlation of activity within events. We compared this to a null distribution computed in the same way but with 1000 iterations of shuffled event boundary locations (maintaining event durations). Median pattern similarity within behavioural events and the null event distribution was submitted to a 2 Age (between-subjects; old, young) by 2 Boundary type (within-subjects; behavioural-model event boundaries, null-distribution of behavioural events) ANOVA. This revealed the expected main effect of event, such that pattern similarity was higher within behaviourally-defined events (*M* = .973, *SD* = .012) than the null distribution (*M* = .953, *SD* = .019), *F*(1, 55) = 209.8, *p* < .001, *η^2^_p_* = .792. The main effect of age was not significant, *F*(1, 55) = 0.414, *p* = .523, *η^2^_p_* = .007, nor was the 2-way interaction between age group and event, *F*(1, 55) = .675, *p* = .415, *η^2^_p_* = .012. Thus, pattern similarity was higher within behaviourally-defined events than would be expected by chance, and this effect did not differ between age groups.

**Boundary transitions.** To determine whether these behaviourally-defined events captured neural activity shifts at their boundaries, we also assessed whether pattern similarity was more distinct in the two seconds immediately surrounding boundaries than the null distribution of shuffled boundary locations. We then submitted these to the same 2 Age (between-subjects; old, young) by 2 Boundary type (within-subjects; behavioural-model event boundaries, null-distribution of behavioural events) ANOVA. For the time directly around these behaviourally defined boundaries (median of the average pattern similarity in the 2 seconds surrounding each boundary), there was no significant main effect of boundary vs. non-boundary timepoint, though there was a trend in the opposite direction compared to what would be expected, with numerically higher pattern similarity immediately around behaviourally-defined boundary timepoints, *f*(1, 52) = 3.90, *p* = .054, η^2^*_p_* = .070. There was no interaction (*p* = .113), nor a main effect of age (*p* = .172). This suggests that the moments immediately surrounding behaviourally-defined event boundaries did not differ from the null distribution of shuffled events.

Together, these results are consistent with the notion that the behaviourally-derived events represent meaningful ongoing states of the film content that have distinct neural representations on average, but may not align precisely enough across participants to be captured in boundary transition analyses. This reinforces the notion that there are important individual differences in boundary timing that are not captured by group level behavioural boundary identification.

**Parietal GSBS**

We tested whether the effects observed at frontal sites would replicate at temporal and parietal sites which have also been shown to play a role in event segmentation (Baldassano et al., 2017; Silva et al., 2019). Though some work suggests that neural state segmentation may occur at a faster timescale in these regions (Sava-Segal et al., 2023), there is some evidence that neural state boundaries in these posterior regions align more strongly across individuals than those at frontal sites (Sava-Segal et al., 2023). We therefore tested whether the alignment of boundaries obtained from running GSBS on parietal sites would align across individuals and with behaviourally-derived event boundaries. Further, to replicate our analyses at frontal sites, we tested whether pattern similarity around neural state boundaries determined through application of GSBS to parietal sites, or interindividual alignment of these boundaries would predict memory performance. To this end, the GSBS algorithm was run in the exact same manner as described in the main text but with 29 temporal and parietal electrodes (approximating the positions of Pz, P1, P3, P5, P7, P9, T7, TP7, CP3, CP5, P2, P4, CP2, P10, P8, P6, CP6, CP6, CP4, T8, TP8; see Supplementary Figure 2).


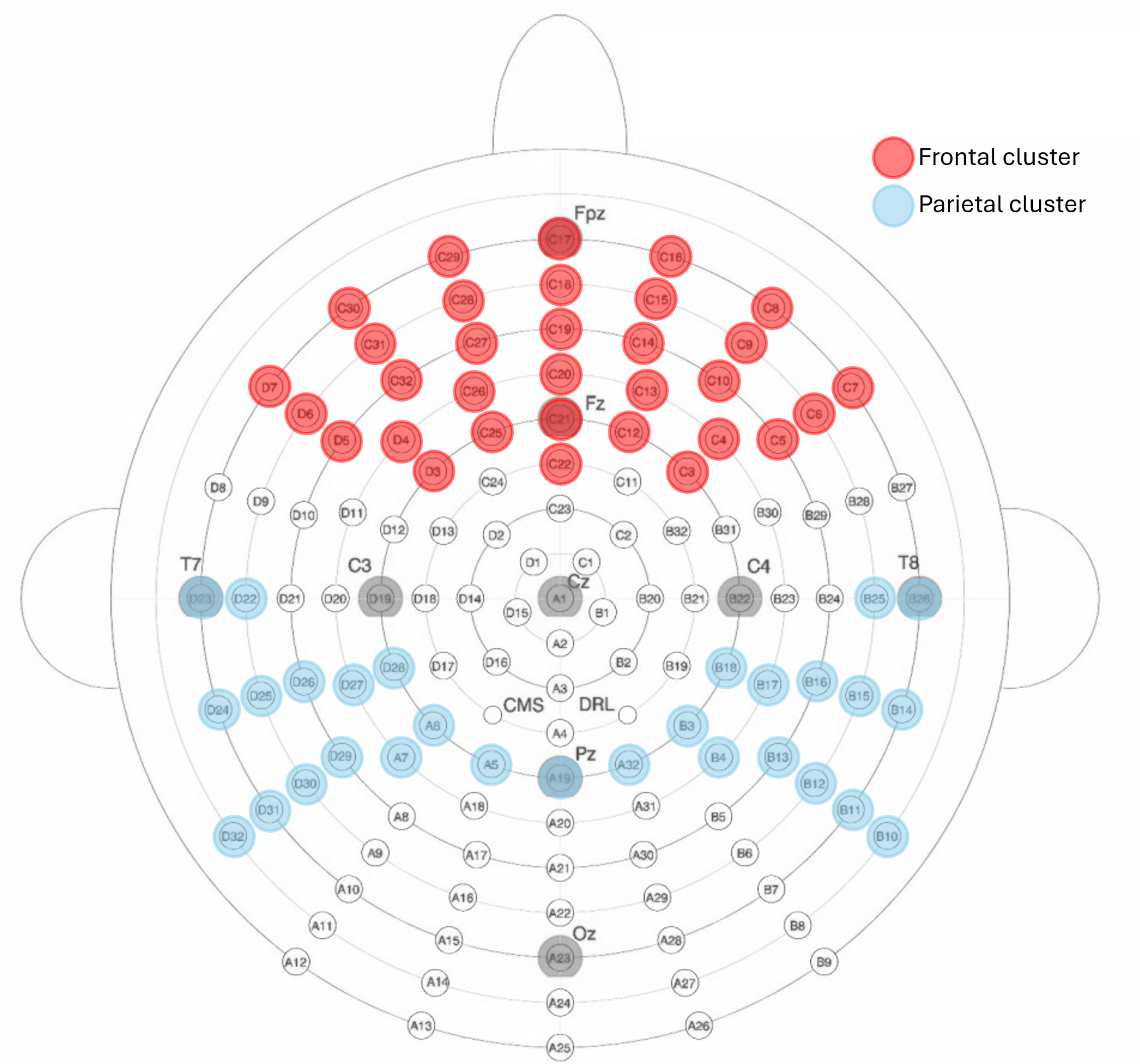


*Supplementary Figure 2*. Electrode locations used in analyses for frontal and parietal clusters from BioSemi 128 channel montage.

***Between-subject State Alignment***

The association between individual and group GSBS boundaries (averaged across all participants) was significantly above chance in both younger (*M* = .238, *SD* = .138), *t*(27) = 9.12, *p* < .001, *d* = 1.72, and older adults (*M* = .255, *SD* = .163), *t*(25) = 8.01, *p* < .001, *d* = 1.57 (see Supplementary Figure 3 for the distribution of adjusted match statistics within each age group). An independent samples t-test revealed that there was no age difference in the average correlation to the group, *t*(52) = .421, *p* = .675, *d* = .115. Thus, replicating effects at frontal sites, GSBS identified neural state changes at parietal sites that significantly aligned across participants, and this alignment did not differ with age.


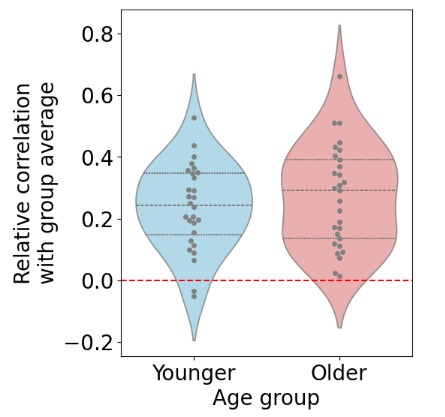


*Supplementary Figure 3.* Correlation between each individual and the group-level boundary density (computed in a leave-one-out manner) at a parietal cluster adjusted for the maximum possible correlation (based on number of boundaries) in each individual. More positive values reflect greater alignment with the group.

***Individual GSBS Alignment with Behaviourally-defined Boundaries.***

We also assessed the association between behaviourally defined events and individual GSBS neural state boundary locations. This alignment was not significantly greater than the similarity to shuffled boundary locations in the overall sample (*M* = -.019, *SD* = .127), *t*(53) = -1.10, *p* = .274, *d* = -.146. An independent samples t-test revealed no age difference in the average association with behaviourally identified boundaries (averaged across all participants), *t*(52) = 1.18, *p* = .244, *d* =.321. Thus, neural states identified using GSBS did not significantly align with the location of event boundaries identified by a separate group of participants engaging in intentional boundary identification.

**Parietal GSBS association with memory performance**

**Between-subject State Alignment.** We evaluated whether individual differences in alignment to the whole group would predict memory performance, with the expectation that we would replicate the observed frontal effect that alignment to the group would not predict memory performance. A linear regression was run predicting the proportion of internal details recalled from the adjusted correlation between each individual and their group, the individuals’ age group, and the interaction between these two factors. Within this model, age group was not a significant predictor of memory performance (β = .046, *p* = .127), an individual’s correlation with the group did not predict memory performance (β = .076, *p* = .358), and the two-way interaction was not significant (β = -.047, *p* = .679). This replicates the frontal site results and suggests that alignment with the group did not predict memory performance (see Supplementary Figure 4A for regression lines for each age group and individual data points).

**Pattern Similarity Around State Boundaries.** We assessed whether pattern similarity around state boundaries at parietal sites would predict memory performance, with the expectation that a greater change in the pattern of activity at state boundaries (i.e., more distinct boundaries) should relate to better memory for the movie, replicating the effects observed at frontal sites. A linear regression was run predicting the proportion of internal details recalled from the relative neural pattern similarity in the 2 seconds surrounding neural state boundaries (adjusted for the similarity in the center of neural states), age group, and the interaction. Within the model, age group significantly predicted memory performance (β = .066, *p* = .006), such that younger adults performed better on average. The relative similarity around neural state boundaries also predicted memory performance (β = -1.80, *p* = .020), such that lower correlations around GSBS boundaries (i.e., more distinct boundaries) predicted higher memory performance. The interaction between age group and pattern similarity around boundaries was not significant (β = 1.56, *p* = .166). Thus, similar to the frontal results, memory performance was higher for individuals with more distinct boundaries as reflected by lower correlation of neural activity in the two seconds before and after a state boundary and this effect did not differ according to age group (see Supplementary Figure 4B for regression lines for each age group and individual data points).

Thus, across both analyses we replicated the critical findings observed at frontal sites using the temporal-parietal cluster. Pattern distinctiveness around neural state boundaries predicts better memory performance while interindividual alignment between state boundary locations does not.


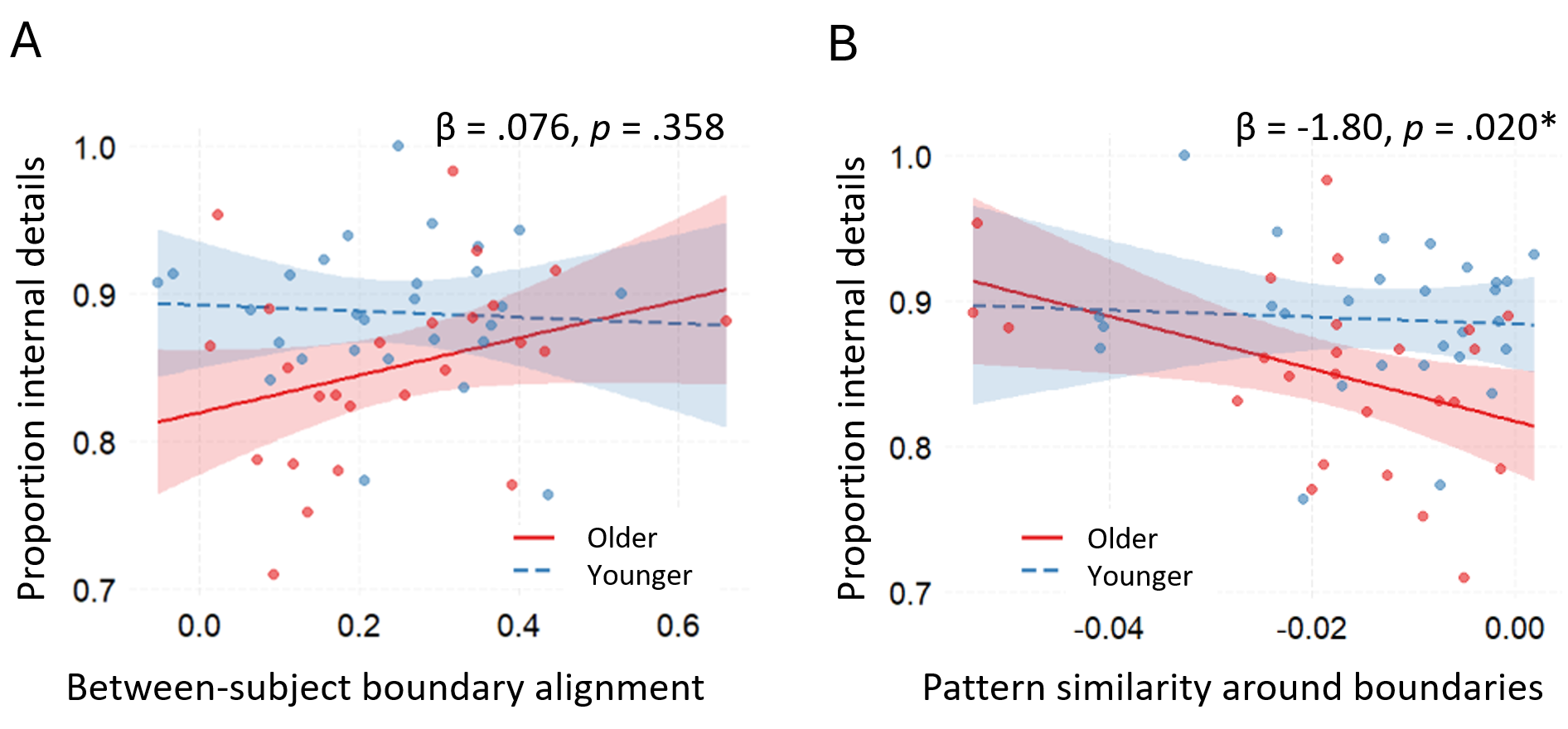


*Supplementary Figure 4.* Memory performance predicted by between-subject state alignment and pattern similarity around state boundaries at parietal sites. Proportion of internal details calculated as the number of internal details (reflecting some aspect of the movie) divided by the total number of details provided during free recall. (A) Between-subject boundary alignment calculated as similarity to all other individuals’ neural state boundary placement adjusted for the maximum possible alignment for each individual. (B) Pattern similarity in the two seconds surrounding neural state boundaries adjusted for individual’s pattern similarity in the center of each neural state. Shaded areas represent 95% CI.
